# The Interaction of Slaughtering, Drying, and Defatting Methods Differently Affects Oxidative Quality of the Fat from Black Soldier Fly (*Hermetia illucens*) Larvae

**DOI:** 10.3390/insects14040368

**Published:** 2023-04-08

**Authors:** Raúl Hurtado-Ribeira, Diego Martín Hernández, David Villanueva-Bermejo, Mónica R. García-Risco, M. Dolores Hernández, Luis Vázquez, Tiziana Fornari, Diana Martin

**Affiliations:** 1 Departamento de Producción y Caracterización de Nuevos Alimentos, Instituto de Investigación en Ciencias de la Alimentación (CIAL) (CSIC–UAM), C/Nicolas Cabrera 9, Cantoblanco Campus, 28049 Madrid, Spain; 2 Sección Departamental de Ciencias de la Alimentación, Facultad de Ciencias, Universidad Autónoma de Madrid, 28049 Madrid, Spain; 3 Instituto Murciano de Investigación y Desarrollo Agrario y Alimentario (IMIDA), Estación de Acuicultura Marina, Puerto de San Pedro del Pinatar, 30740 Murcia, Spain

**Keywords:** edible insects, killing, fat extraction, lipids, insect meals, oxidative stability, antioxidant

## Abstract

**Simple Summary:**

In addition to the potential of edible insects as an alternative source of proteins, the production of insect meals generates a significant amount of fat that is also of interest for food and feed. The consideration of the impact of processing factors on the quality of insect fats, such as oxidative stability, is indispensable to fully realize their value as a co-product. Therefore, this study aimed to understand how the different successive modes of processing of *Hermetia illucens* larvae (black soldier fly larvae, BSFL), such as slaughtering (by blanching or freezing), drying (by oven-drying or freeze-drying) and defatting (by mechanical pressing or supercritical CO_2_ SFE), as well as their interactions, affect the oxidation of the resulting fat and defatted meal. The preferred procedure would be a combination of freezing with freeze-drying and mechanical pressing, especially the combination of freeze-drying and mechanical pressing, for the best oxidative quality during storage. Such oxidative stability was strongly related to the antioxidant activity of the fats, which was conditioned by the processing methods used for BSFL. Therefore, this study provides guidance on selecting the best combinations of slaughtering, drying, and defatting processes for BSFL to obtain fats with the best oxidative quality.

**Abstract:**

The interrelation effect of slaughtering, drying, and defatting methods of BSFL on the oxidative quality of the derived fat was studied. Blanching and freezing were compared as slaughtering methods, followed by oven or freeze-drying for drying and mechanical pressing or SFE for defatting. The oxidative state and stability of the extracted fat and defatted meals were monitored immediately after their production, using peroxide value (PV) and Rancimat test, and over 24 weeks of storage. Slaughtering and drying methods had an independent effect on PV, with freezing and freeze-drying being the best methods. Mechanical pressing and SFE were comparable and superior to conventional hexane defatting. Interactions were observed between slaughtering and defatting, drying and defatting, and between all three factors. Generally, freeze-drying combined with any of the slaughtering and defatting methods resulted in the lowest PVs, with mechanical pressing being preferred. Freeze-drying plus mechanical pressing also produced the most stable fats during storage according to the evolution of PV, while the combination of blanching and SFE produced the least stable. A significant correlation was found between the PV at 24 weeks and the antioxidant activity of the fats. Contrary to storage assays, in accelerated Rancimat assays, freeze-dried samples were the least stable, which was partially attributed to the significant correlation with the acid values of the samples. Defatted meals followed a similar pattern to the extracted fat, except for worse oxidation for SFE defatting. Therefore, the different processing methods of slaughtering, drying, and defatting of BSFL differently affect lipid oxidation, with interactions between such successive steps.

## 1. Introduction

The potential of edible insects as an alternative source of protein for food and feed is widely recognized. Since 2021, the European Union has authorized the placing on the market of up to four edible insect species, *Tenebrio molitor* (mealworm, edible life stage as larva), *Locusta migratoria* (migratory locust, edible life stage as an adult), *Acheta domesticus* (house cricket, edible life stage as adult) and *Alphitobius diaperinus* (lesser mealworm, edible life stage as larva) for human consumption [1,2,3,4]. It is expected that in the coming months, EFSA will issue more favorable opinions on requests made for other insect species, such as *Hermetia illucens*, *Gryllodes sigillatus*, or *Apis mellifera*. However, since 2017, most of these insect species have already been authorized for feed production for aquaculture [5] and, since 2021, for poultry and pig feeding [6]. Among these insect species, the larvae of *Hermetia illucens* (black soldier fly larvae, BSFL) are one of the most popular and promising sources of alternative protein for feed due to their high potential and positive results, along with their great bioconversion ability of food waste into a value product [7,8,9]. BSFL has a protein content ranging from 36 to 65%, high lipid content in the range of 5–40%, and is rich in chitin and micronutrients [7,8].

As in any process of food and feed manufacturing, the technological processing of raw materials can directly impact the quality, nutritional composition, digestibility, or palatability. These aspects are even more particular when using raw materials of animal origin, such as edible insects. This is because, unlike materials of plant origin, a stage of slaughtering of these animals is necessary, and subsequent microbiological and chemical stabilization is later required through a reduction in water activity by drying [10]. Finally, defatting is also a common step for the use of edible insects to produce high-protein products, which is currently the primary purpose and interest in using edible insects. Thus, defatting is almost mandatory for insects with high lipid content, such as those consumed in the larval stage, like BSF, which can have lipid contents up to 40% [11]. Lipids dilute protein concentration and, in the case of feed, may hinder manufacturing technology, impact palatability, and affect productive yields and lipid composition of animals.

In addition to the indisputable value of insect proteins, it is important to note that the defatting process of insects produces a significant amount of fat that also has great potential for use in food and feed due to the attractive fatty acid profile of many species and the presence of minor lipid compounds of interest [12,13]. For example, in the case of BSFL, its fat is gaining popularity due to its fatty acid profile, which is mainly composed of lauric acid, accounting for approximately 50%, a ratio similar to that found in coconut oil [14]. Lauric acid is a well-known fatty acid with interesting properties for food, cosmetic and pharmaceutical applications [15,16]. Palmitic acid, myristic acid, oleic acid, linoleic acid, and α-linoleic acid are also typical fatty acids found in BSFL. Therefore, despite being mainly saturated fat (>70%), it can contain values of up to 10% polyunsaturated fatty acids and over 20% unsaturated fatty acids. Thus, as for other food lipids, the consideration of factors related to the quality of these new lipids from edible insects, such as oxidative quality and stability, is indispensable. This is because lipid oxidation is a major cause of quality deterioration in food products, leading to modifications in flavor and nutritional quality. However, information regarding the oxidative stability of edible insect lipids is not always a primary focus in studies, given the greater interest in proteins. However, in this sense, it should be noted that complete defatting of insect meals is not always achieved; hence, the lipid oxidation of the residual fat content of the defatted products may also impact the quality and deterioration of the insect meals.

It is important to note that the three main processes indicated for the processing of insect larvae (slaughter, drying, and defatting) can have a significant impact on the lipid oxidation of the fats and meals. Especially thermal methods, pro-oxidant conditions, or degradation of minor antioxidant compounds due to the processing can all contribute to lipid oxidation [17,18,19], while non-thermal methods may also favor enzymes involved in lipid oxidation [19]. Concerning slaughtering, blanching, and freezing are the most popular methods [18]. For example, after testing different killing methods, Larouche et al. (2019) [17] and Zhen et al. (2020) [19] remarked blanching as the preferred method for all the quality parameters of BSFL meals, including low lipid oxidation. Concerning drying methods, either thermal methods such as oven or microwave, as well as non-thermal methods such as freeze-drying, have been tested [20,21]. However, no conclusive results have been reached regarding the impact of the drying methods on lipid oxidation, likely due to the dependence on the slaughtering method used. Finally, concerning defatting, mechanical pressing or solvent extraction are the most popular methods, but other advanced and sustainable technologies are emerging, such as lipid extraction by supercritical fluids (SFE-CO_2_) [11]. However, despite the relevance that this final step of insect meal processing might also have on lipid oxidation, this has not been fully explored, likely because the insect meal quality is the main aim after the defatting step. Therefore, conclusive results regarding the impact of the slaughtering, drying, and defatting processes of BSFL on lipid oxidation have not been reached, and further studies are necessary to fully characterize and value this co-product obtained during the production of insect meals. Additionally, one of the most important questions to consider is that the current scarce studies on lipid oxidation of insect lipids have only examined the impact of one or two of the process steps in isolation, namely slaughtering or drying, or defatting. However, the consideration of the three technological processes together and the interrelation between them as successive operations of a single final process for obtaining valuable insect meals and fats should be approached.

Therefore, the main aim of this study was the exploration of the impact and interrelation of different slaughter, drying, and defatting conditions of BSFL on the oxidative quality and oxidative stability of the obtained fat as the main coproduct of insect meal production. To perform this study, blanching and freezing were compared as slaughtering methods, each of them were followed by oven or freeze-drying as drying methods, and each of them were followed by mechanical pressing or SFE-CO_2_ as defatting methods. Oxidative quality and stability of both the extracted fats and defatted meals were monitored immediately after the production, as well as during a storage period of 6 months, by monitorization of lipid peroxidation as peroxide value. Accelerated oxidation conditions were also tested for the extracted fat.

## 2. Materials and Methods

### 2.1. Slaughtering of Larvae

Larvae of BSF were reared and slaughtered by Entomo Agroindustrial (Cehegin, Spain) by blanching or freezing, with each procedure being performed in duplicate (8 kg per replicate). Prior to slaughtering, the larvae were sifted and washed by immersing them in a basket in cool water and then drained. For blanching, the larvae were immersed in water at 90 °C at a ratio of 1:10 (*w*/*v*) of sample to water for 40 s. After that, the larvae were immersed in cool water and drained. For freezing, the larvae were frozen at −20 °C for 24 h. After each slaughtering procedure, each batch was divided in half to be dried by oven or freeze-drying.

### 2.2. Drying of Larvae

Oven drying was carried out at 65 °C for 24 h in a conduction oven in trays with a maximum thickness of larvae layer ≤ 1 cm. Freeze-drying was performed for 4 days using a 3-tray freeze drier (LyoBeta 15, Telstar, Terrassa, Spain) with a program of −20 °C for 2 h, followed by a gradual increase from −20 to 20 °C, and then maintained at 20 °C for the remainder of the time, with the condenser at −81 °C. After each drying procedure, each batch was divided in half to be defatted by either mechanical pressing or SFE. The dried samples were stored at room temperature and defatted as soon as possible.

### 2.3. Defatting of Samples

For mechanical defatting, 450 g of dried larvae were extracted using a screw-press expeller (InVIA, Barcelona, Spain) with a heating jacket to allow the drainage of the oil. The press was pre-heated at the minimum temperature (136.0 ± 13.1 °C), and the temperature of the press head was continuously monitored during pressing, reaching a mean maximum value of 140.4 ± 10 °C and a mean minimum value of 126.6 ± 11.9 °C, depending on the batches. The collected crude fat was centrifuged (Multifuge 3SR+ centrifuge, Thermo Scientific, Waltham, MA, USA) at 3400× *g* for 10 min to remove co-extracted solids.

Supercritical CO_2_ defatting was performed using supercritical CO_2_ extraction equipment (Model Thar SF2000, Thar Technology, Pittsburgh, PA, USA) and following the optimized conditions previously performed by Cantero-Bahillo et al. (2022) [11]. Prior to extraction, 500 g of dried larvae were ground in a knife mill (Grindomix GM 200, Retsch GmbH, Haan, Germany). The extraction cell (1350 cm^3^) was loaded with 450 g of sample. The defatting was performed at 450 bars, 60 °C, and CO_2_ flows of 130 g/min for 240 min. For comparative purposes with the traditional method of defatting by solvents, hexane was also used, according to Cantero-Bahillo et al. (2022) [11]. Thus, 2 g of ground sample were homogenized with hexane at a ratio of sample to solvent of 1:5 (*w*/*v*) in an Ultraturrax (Ultra-turrax T18 basic, IKA, Staufen, Germany) (11,000 rpm) for 5 min. Then, the mixture was centrifuged at 4500 rpm for 10 min at 20 °C. The supernatant was removed, and the precipitate was defatted again following the same procedure. Hexane was removed using a vacuum rotary evaporator.

For each treatment of combined methods of slaughtering, plus drying and defatting, duplicated samples of fat and defatted meals were obtained.

The initial fat content of the dried samples varied in the range of 18–29% (using the described hexane method for defatting). After defatting, values of fat yield in the range of 9–21% were obtained, depending on the combined treatments of slaughtering, drying, and defatting. In the case of the defatted meals, values of remaining fat were in the range of 1–10%, also depending on the processing methods.

For all the defatting procedures, the lipid samples and defatted meals were immediately used for oxidative measurements.

### 2.4. Oxidative Stability by Rancimat Test

Lipid samples (2 g) were subjected to accelerated oxidative conditions by a Metrohm Rancimat model 743 (Herisau, Switzerland). The samples were oxidized at an airflow rate of 20 L/h and 120 °C. The changing of the conductivity (µS/cm) of the distilled water cell (60 mL) as a consequence of the solubilization of volatile acids formed in the samples due to the forced oxidation was monitored over time. The results were expressed as the induction time (IT), which was automatically determined as the inflection point of the generated plot of conductivity of the water versus time (h). The analysis was performed in duplicate.

### 2.5. Peroxide Value

The peroxide value (PV) of lipid samples was immediately measured after being obtained using the photometric FoodLab instrument (CDR S.r.L., Ginestra Fiorentina), according to Martin et al. (2012) [22]. This method, equivalent to the AOCS Official method Cd 8–53, is based on a rapid photometric reaction of 5 µL of sample added by a positive displacement pipette to 1 mL of the reaction reagent of the supplier in prefilled cuvettes. After adding the sample, 10 μL of the second reagent of the supplier was added. The mixture was reacted for 3 min at 37 °C in the incubation cells of the thermostated instrument. The samples were then measured at 505 nm in the measuring cells, with a measuring range for quantification given from 0.1 to 50 mEqO_2_/kg, using the default settings for calibration and quantification.

For storage experiments, two samples of 4 g of fat from each batch were stored in screw-capped glass tubes of 4 mL at room temperature (20 ± 2 °C) in the dark. PV was periodically measured for 24 weeks. Each sample was measured in duplicate. Samples were purged with nitrogen prior to storage and after each measurement.

For defatted meals, samples of 50 g were stored in screw-capped flasks of 100 mL at room temperature in the dark. The PV of the residual fat was periodically measured for 24 weeks after extraction with hexane as described above and in duplicate.

### 2.6. Statistical Analysis

To assess the effect of the combined methods of slaughtering, plus drying and defatting, two independent sets of duplicated samples were obtained for each treatment, and each replicate of the sample was analyzed in duplicate. The analysis results were treated as if they were four replicates of one unique sample, based on the negligible variability observed for the sample replicates, according to the low deviation obtained for the results. This approach allowed for minimizing the number of samples required while still achieving sufficient statistical power to detect differences between treatments. The effect of considered factors (slaughtering, drying, and defatting procedures) and their respective interactions on oxidative stability was evaluated by a three-way analysis of variance using the general linear model procedure of the SPSS 26.0 statistical package (SPSS Inc., Chicago, IL, USA). When the effect of any of the factors was significant (*p* ≤ 0.05), differences between groups were analyzed using the post-hoc Tukey’s tests.

## 3. Results and Discussion

### 3.1. Oxidative State of the Fat after Extraction

**Figure 1:**
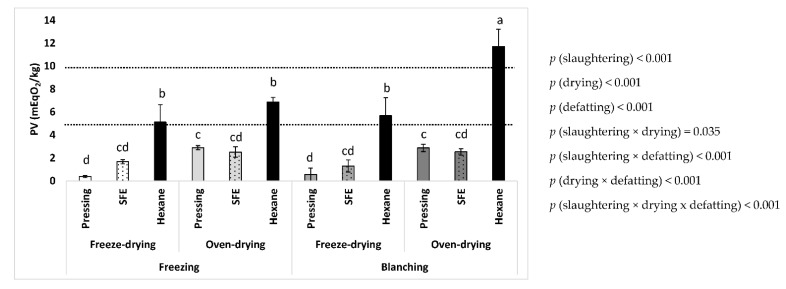
Initial oxidative state (PV) of the fat from BSFL processed by different methods of slaughtering, drying, and defatting. Dotted line means maximum reference values. Different letters between the treatments mean significant differences. Each data point corresponds to mean values ± SD, n = 4.

The PV of fat samples of BSFL obtained after different modes of slaughtering, drying, and defatting were measured immediately after production. Significant effects of the three tested factors as well as their interactions on the initial oxidative state of the fats, were obtained (Figure 1). Concerning the effect of slaughtering, freezing produced fats with a better initial oxidative state compared to blanching (3.2 and 4.1 mEqO_2_/kg, respectively, as mean values regardless of the method of drying and defatting). With respect to the drying method, the samples from freeze-drying showed better oxidative quality compared to oven-drying (2.5 and 4.9 mEqO_2_/kg, respectively, as mean values regardless of the method of slaughtering and defatting). Therefore, the thermal procedures of slaughtering and drying caused worse oxidation of the obtained fats, both as individual steps and when combined (*p* [slaughtering × drying] < 0.05). Consequently, the lowest mean values were obtained when slaughtering by freezing and drying by freeze-drying (2.4 mEqO_2_/kg, mean value regardless of defatting method). In contrast, the PV increased more than doubled when slaughtering by blanching and drying by oven-drying (5.7 mEqO_2_/kg, mean value regardless of defatting method).

Finally, concerning the method of defatting, it was clearly observed a worse oxidation of samples from hexane defatting, while the oxidative state from mechanical pressing and SFE was much lower and comparable between them (7.3, 1.7 and 2.0 mEqO_2_/kg, respectively, as mean values regardless of the method of slaughtering and drying). Interestingly, there were significant interactions between the defatting and the slaughtering methods, the defatting and the drying method, as well as between the three variability factors. Thus, the lowest oxidative value was obtained for the combination of freeze-drying and mechanical pressing, while the combination of blanching, oven-drying, and hexane resulted in the highest PV (11.7 mEqO_2_/kg).

Currently, there are no specific regulations or standards for the quality parameters of oils and fats derived from edible insects, including those related to oxidation. Therefore, in the absence of such standard values, it should be recommended to keep the oxidation levels as low as possible. This is because a repeated intake of oxidized oils can be harmful to humans and animals. Furthermore, given that the current main use of BSFL is for animal feed, mainly aquaculture, it should be taken into account that diets with high PV might cause damage to fish, such as oxidative stress, inflammation, negative effect on immune response and on antioxidant capacity, oxidative muscle damage, as well as a decrease in intestinal microbiota diversity, among other pathological changes [23,24,25]. Due to this evidence, the established maximum PV values for fish oil [26] and for edible oils (specifically, those edible fats and oils not covered by individual standards) [27], which are 5 and 10 mEqO_2_/kg, respectively, could be considered as reference points of oxidative quality. We referenced either the general value of edible oils, but also the low value of fish oil because, despite being quite different form fish oil, the general use of BSFL is being proposed for animal feeds, including aquaculture feeds, which are mainly based on fish oils. Therefore, for comparative purposes, the reference for fish oil was taken into account. Nevertheless, for BSFL, we consider proper the reference value of 10 mEqO_2_/kg given by the International Food Standards of Codex Alimentarius for the general group of “edible oils” [27]. This is because, in the absence of specific regulations for edible insect oils, we consider that this is the current standard that best adjusts to the case of insect oils. According to Figure 1, all fats obtained from defatting by mechanical pressing or SFE had PV values below the reference values, ranging from 0.4 to 2.9 mEqO_2_/kg, suggesting good oxidative quality, regardless of the method of slaughtering and drying. In contrast, all those samples produced by hexane defatting exceeded the reference PV of 5 mEqO_2_/kg, with some even exceeding 10 mEqO_2_/kg, especially those obtained from slaughtering by blanching and drying by oven-drying. This last result could be explained by a combined effect of pro-oxidant factors, such as thermal processing of slaughtering and drying, along with the conditions of hexane defatting, which include extended air exposure during defatting. Due to the poor oxidative state of the hexane samples, they were excluded from subsequent assays. Therefore, the obtained results suggest that both mechanical pressing and SFE would be more respectful procedures for defatting than the conventional solvent process by hexane, regardless of the method of slaughtering and drying.

Previous data on the PV of BSFL fat are limited, and the impact of the interrelation between the three major processing stages has not been thoroughly investigated. For example, concerning only slaughtering, Larouche et al. (2019) [17] showed comparable lipid oxidation values for freezing and blanching. Matthäus et al. (2019) used vacuum drying for the simultaneous slaughtering and drying of BSFL, followed by fluidized bed drying and mechanical pressing for defatting. These authors reported a PV of 0.29 mEqO_2_/kg [28]. This value was similar to that observed in the present study after freeze-drying and mechanical pressing for defatting. Mouithys-Mickalad et al. (2021) reported the PV of BSFL fat obtained by hexane under stirring for 1 h from commercial BSFL meal but, unfortunately, did not provide information about the processing conditions of the larvae used to produce the BSFL meal. These authors described a PV of approximately 3.5 mEqO_2_/kg for the extracted fat [29].

### 3.2. Oxidative Stability of the Fat by Rancimat Method

**Figure 2:**
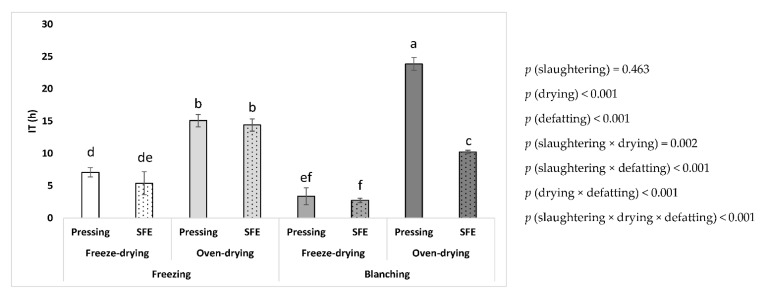
Oxidative stability (IT) by the accelerated Rancimat method of the fat from BSFL processed by different methods of slaughtering, drying, and defatting. Different letters between the treatments mean significant differences. Each data point corresponds to mean values ± SD, n = 4.

The oxidative stability of the different lipid samples from BSFL immediately after production was measured by the procedure of accelerated oxidation conditions of temperature and air by the Rancimat method (Figure 2).

As shown in Figure 2, drying and defatting and different double and triple interactions significantly affected the induction time of oxidation (*p* > 0.05). However, in the case of slaughtering, a lack of effect of this independent factor was observed (*p* < 0.05). Thus, the oxidative stability of the fats under accelerated conditions remained the same, regardless of whether the samples were frozen or blanched. Concerning the drying process, freeze-drying resulted in samples with worse stability compared to oven-drying (4.6 and 15.9 h, respectively, as mean values regardless of the method of slaughtering and defatting). Therefore, this result is opposite to what was observed for the initial oxidative state of the samples, where the samples from freeze-drying showed the best initial values of oxidation compared to oven-drying (Figure 1). Concerning the method of defatting, those obtained through SFE were less stable than those obtained through mechanical pressing (8.2 and 12.3 h, respectively, as mean values regardless of the method of slaughtering and drying). Again, this result was not in agreement with the initial PV of samples, which showed the same initial value of oxidation for SFE and mechanical pressing (Figure 1). Regarding the interactions between the factors, all the two-factor and three-factor interactions were significant. Thus, the worst oxidative stability was found when the combination of blanching, freeze-drying, and SFE was performed (2.7 h), while the best stability was obtained when the samples were subjected to the combination of blanching, oven-drying, and mechanical pressing (23.9 h).

**Figure 3:**
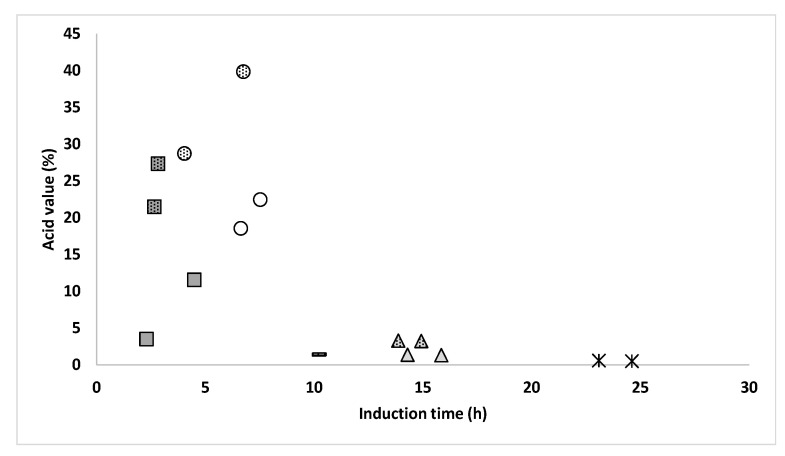
Correlation between induction time after Rancimat test (h) and acid value (%) of the fat from BSFL processed by different methods of slaughtering, drying, and defatting. Undotted circles (freezing, freeze-drying, mechanical pressing), dotted circles (freezing, freeze-drying, SFE), undotted triangles (freezing, oven-drying, mechanical pressing), dotted triangles (freezing, oven-drying, SFE), undotted squares (blanching, freeze-drying, mechanical pressing), dotted squares (blanching, freeze-drying, SFE), asterisks (blanching, oven-drying, mechanical pressing), line (blanching, oven-drying, SFE).

In the general field of oils and fats, it is important to note that the initial oxidative state does not necessarily determine the later oxidative stability. Stability can be marked by other factors such as the fatty acid profile, lipid composition, or the presence of minor antioxidant or pro-oxidant compounds in the sample [22]. With respect to fatty acids, all the samples exhibited the same general profile, regardless of the method of slaughtering, drying, and defatting. Therefore, this factor would not determine the differences in oxidative stability under induced accelerated conditions. Regarding lipid composition, it is known that one factor that might lead to worse oxidative stability is the content of free fatty acids (FFAs) since these species are more prone to oxidation than esterified fatty acids [30,31]. To test this hypothesis, the acid value of the samples (measured by the volumetric titration method according to ISO 660:2020 [32]) was used to establish a Pearson’s correlation test with the induction time (Figure 3). As shown in Figure 3, the different lipid samples exhibited a wide range of acid values. High levels of FFAs in insect lipids have been linked to the hydrolysis of triglycerides by endogenous lipases [29]. Such enzymes will act more intensively when inhibitory conditions are lacking during processing, mainly through non-thermal methods [17]. This would explain why the highest acid values were measured for samples from slaughtering by freezing and drying by freeze-drying, and the lowest values were measured for slaughtering by blanching and drying by oven-drying (Figure 3). A significant negative correlation was found between the oxidative stability and the acid value (*r* = −0.636, *p* = 0.008). Therefore, this suggested that the higher the acid value, the lower the oxidative stability. However, this relation was not linear, and two principal groups of samples could be identified in Figure 3, namely those from oven-drying with higher induction times and lower acid values and those from freeze-drying with lower induction times and higher acid values. Thus, in general, it could be said that for acid values ≤ 3%, the samples showed the highest induction times, being ≥ 10 h, but up to values around 25 h, whereas for acid values >3%, the samples showed induction times ≤ 10 h, ranging up to values around 2 h (Figure 3). Therefore, this non-linear correlation between acid value and oxidative stability would only partially explain the differences in induction time between samples, and the dispersion of data in Figure 3 suggests that other differences between the composition of the samples are involved in their different oxidative stability under accelerated Rancimat conditions.

The available information on the oxidative stability of BSFL fat is limited, but some data are beginning to emerge. Matthäus et al. (2019), that processed BSFL by vacuum drying for slaughtering, fluidized bed for drying, and mechanical pressing for defatting, described induction times of oxidation for the fat after Rancimat oxidation of 50 h, along with an FFA value of 0.98% [28]. Recently, Nekrasov et al. (2022) described values > 48 h for the Rancimat test but under non-comparable Rancimat conditions at 100 °C instead of 120 °C of the current study. They used BSFL dried under a non-specific method and defatted by mechanical pressing [33]. Xu et al. (2021) reported values of induction time of 14.6 h of fat from BSFL but did not describe the mode of processing of the larvae [34].

### 3.3. Oxidative Stability of the Fat under Storage Conditions

Regarding lipid oxidation, it is important to note that it undergoes different reaction pathways depending on temperature, which can affect the solubility of oxygen as well as the stability of potential antioxidants or pro-oxidant compounds [35]. As a result, the prediction of oxidative stability based on accelerated tests performed at high temperatures, such as the Rancimat method, has some limitations, which have been pointed out as one of the issues with this method [22,35]. Hence, depending on the type of oil, predictions may range from acceptable estimation to overestimation or underestimation [22,36]. Consequently, in the study of the oxidative stability of novel edible oils, such as BSFL fat, more extensive assays, including storage experiments of oxidation under normal storage conditions, are necessary. To the best of our knowledge, previous studies in this regard have not been reported in the scientific literature for BSFL fat. For this study, samples of fat obtained immediately after extraction were stored at room temperature, in the dark, and in the absence of air, and changes in PV were monitored for 24 weeks. We only considered, the changing in primary oxidation as PV because, dalthough other parameters related to secondary oxidation could also be produced and varied during storage, PV is currently the only standardized recommended parameter of oxidative quality for edible oils [27], and the aim of this assay was to establish the loss of oxidative quality of the fat samples over the storage time.

**Figure 4:**
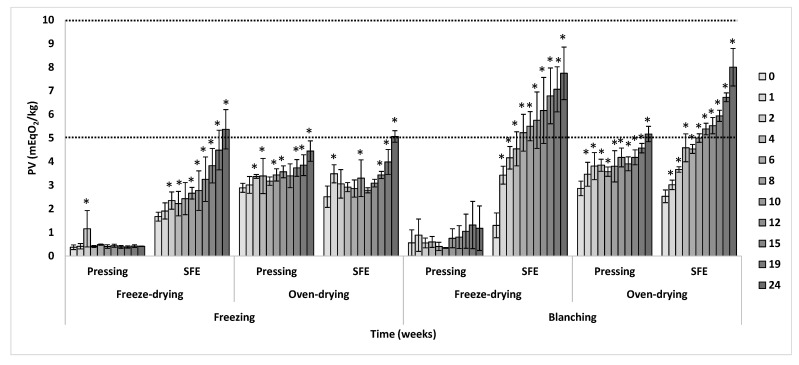
Oxidative stability (PV) during storage of the fat from BSFL processed by different methods of slaughtering, drying, and defatting. Samples with an asterisk within each treatment mean significant difference with respect to its respective initial value at week 0. Each data point corresponds to mean values ± SD, n = 4.

As shown in Figure 4, most of the samples progressively oxidized over time, according to the increasing values of PV, but with varying rates. In general, with respect to the slaughtering, those samples from freezing oxidized at a lower rate compared to those from blanching, suggesting that oils from non-thermal slaughtering would be more stable. In fact, regardless of the time of storage, a significant effect of the slaughtering on PV was obtained (*p* [slaughtering] < 0.001). This result was not in agreement with the results obtained from accelerated experiments of oxidation using Rancimat since a lack of difference was obtained in the oxidative stability due to the slaughtering method. Regarding the drying method, the samples from freeze-drying were more stable (*p* [drying] < 0.001, regardless of the time of storage), but this result was highly dependent on the defatting method (*p* [drying × defatting] < 0.001, regardless of the time of storage). Thus, the most remarkable result of Figure 4 was that the combination of freeze-drying with mechanical pressing led to the most stable fats when the evolution of PV was monitored. It is notable that the PV of these fats did not change even after 6 months of storage and extremely differed from the oxidative behavior of the rest of the treatments. Additionally, this result was opposite to the obtained for the Rancimat test, where all the freeze-dried samples showed the worst oxidative stability. These discrepancies between normal and accelerated tests of oxidation highlight the differences in results that can be obtained when oxidative studies are performed under both methods, as previously explained, and emphasize the relevance of conducting storage assays of oxidation to better understand the impact of processing conditions of BSFL on the stability of the fat obtained, at least from the primary oxidation point of view of PV, as marked by the Codex Alimentarius as the oxidative quality of edible oils [27].

Concerning the defatting method, those samples from SFE followed a higher rate of oxidation in comparison to mechanical pressing (*p* [defatting] < 0.001, regardless of the time of storage), especially those obtained from slaughtering by blanching. In fact, a significant interaction effect between slaughtering and defatting was obtained, regardless of the storage time (*p* [slaughtering × defatting] < 0.001). Thus, SFE samples were the first to exceed the reference standard value of 5 mEqO_2_/kg recommended for fish oils after 2 months of storage following slaughtering by blanching, although this occurred after 6 months of storage following slaughtering by freezing. Nevertheless, from the oxidative quality point of view of edible oils and their recommended PV limit of 10 mEqO_2_/kg [27], none of the samples reached such a limit after 6 months of storage. Finally, it is worth noting that there was no interaction between the modes of slaughtering and drying, as well as among the three variability factors (*p* > 0.05).

As previously explained, oxidative stability can be affected by factors such as the lipid profile or the presence of minor antioxidant or pro-oxidant compounds in the sample. In the case of the Rancimat test, the differences observed could be partially explained by the acid value of the samples, as described in Figure 3. However, this difference in acid value was not related to the oxidative stability under normal storage conditions, as no significant correlation between PV at 24 weeks and the acid value was obtained (*p* > 0.05). In fact, the samples obtained through freeze-drying, which had a high acid value (Figure 3), were the most stable during the storage experiment for mechanical pressing but not for SFE (Figure 4). Therefore, this suggests that the level of FFAs, which are more prone to oxidation, may be more relevant under accelerated oxidation conditions, whereas the initial level of acid value may not have a major impact on the oxidation of BSFL fat under storage conditions.

**Figure 5:**
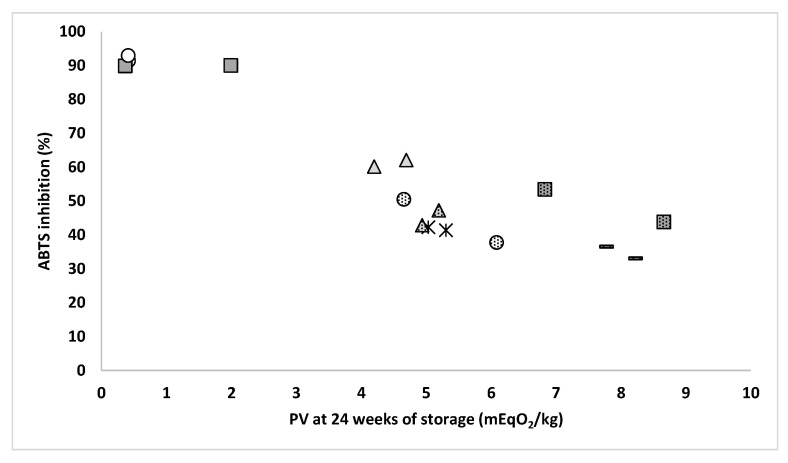
Correlation between the oxidative state (PV) of the different fats from BSFL after 24 weeks of storage time and the initial antioxidant activity (ABST inhibition) of the fat samples. Undotted circles (freezing, freeze-drying, mechanical pressing), dotted circles (freezing, freeze-drying, SFE), undotted triangles (freezing, oven-drying, mechanical pressing), dotted triangles (freezing, oven-drying, SFE), undotted squares (blanching, freeze-drying, mechanical pressing), dotted squares (blanching, freeze-drying, SFE), asterisks (blanching, oven-drying, mechanical pressing), line (blanching, oven-drying, SFE).

It can be hypothesized that the presence or absence of minor antioxidant and pro-oxidant compounds co-extracted during the processing of the larvae oil could be involved in the observed differences in the oxidative stability of fats during storage. In such case, due to the usual thermal lability of minor antioxidants compounds in oils, the impact of these molecules might not be evidenced under accelerated conditions of the Rancimat test, as frequently described for other oils [22,37], which could also explain the discrepancies with the assays of normal storage conditions. Therefore, to understand the likely presence of minor antioxidant compounds in the obtained fats of BSFL as affected by the different processing conditions, the antioxidant ability of the fat samples was determined using the method of ABTS radical scavenging assay of Re et al. (1999) [38]. As shown in Figure 5, a strong significant negative correlation was observed between the inhibition of the ABTS radical and the PV of the samples at 24 weeks of storage (*r* = −0.908, *p* < 0.001). Thus, those samples showing the highest antioxidant ability were the most stable concerning oxidation.

Therefore, it strongly suggests that the superior oxidative stability of the BSFL fat processed by freeze-drying and mechanical pressing could be linked to the presence of minor compounds in these samples with antioxidant activity. Additionally, this finding is of interest in terms of the added value of these samples, as it suggests that the BSFL fat may have antioxidant activity but with extreme variability depending on the mode of larvae processing. Thus, the combination of freeze-drying and mechanical pressing, irrespective of the slaughtering method, would be the most suitable processing conditions to obtain the best antioxidant BSFL fat. Further studies would be of interest to identify the potential antioxidant molecules present in these samples, as well as by using additional methods for measuring antioxidant activity.

### 3.4. Oxidative State and Stability of the Defatted Meals after Production

After processing the larvae by different methods, defatted meals were obtained. However, it is generally accepted that a complete defatting of food samples is rarely achieved. Therefore, a fraction of the remaining fat will be present in the defatted sample. For example, in the current study, a mean remaining fat content of around 7 ± 3% was found in the defatted meals. This lipid fraction will also suffer the typical oxidative deterioration and will impact the quality of the product during the shelf-life of the defatted meal. Therefore, the study of the impact of the insect processing methods on the subsequent oxidative state and stability of the final defatted meals is necessary, considering that defatted protein meal is currently the preferred insect product.

The initial oxidative state of the defatted meals was determined immediately after production by measuring of PV by previously extracting the remaining fat with hexane. In this sense, it should be noted that, as previously shown, the hexane procedure of defatting itself can cause oxidative deterioration of the samples (Figure 1). Therefore, it could be expected that a potential overestimation of the PV of the defatted meals could be obtained. Nevertheless, despite this limitation, a comparable study between processing treatments of larvae could be performed, at least from the qualitative point of view.

**Figure 6:**
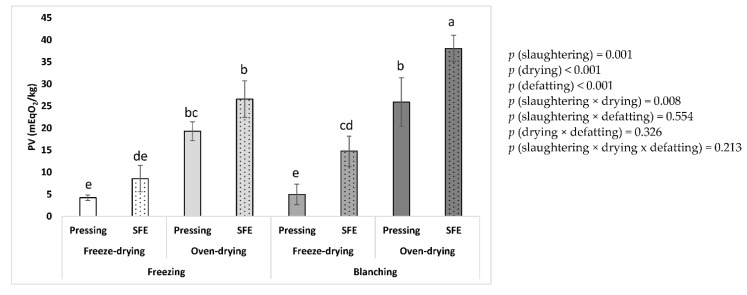
Initial oxidative state (PV) of the defatted meals from BSFL processed by different methods of slaughtering, drying, and defatting. Different letters between the treatments mean significant differences. Each data point corresponds to mean values ± SD, n = 4.

As shown in Figure 6, clear differences were evidenced as a result of the slaughtering, drying, and defatting. Considering the slaughtering, a lower mean value of oxidation was obtained for freezing compared to blanching (14.7 and 20.9 mEqO_2_/kg, respectively, as mean values regardless of the method of drying and defatting). With respect to drying, freeze-dried samples had lower average oxidation than oven-dried samples (8.1 and 27.5 mEqO_2_/kg, respectively, as mean values regardless of the method of drying and defatting).

Therefore, these factors affected the oxidation state of the defatted meals, both individually and in interaction. Thus, regardless of the defatting mode, the combination of freezing plus freeze-drying resulted in the best initial oxidative state of the meals, while blanching combined with oven-drying caused the worst initial PV. Finally, concerning defatting, samples obtained from mechanical pressing showed the best initial PV compared to those obtained through SFE.

The general pattern observed for the effect of the three processing factors agreed, in general, with that observed for the initial oxidative state of the obtained fats after the different procedures (Figure 1). Nevertheless, the evidenced worse oxidation of defatted meals resulting from SFE was not observed for the major extracted fat by SFE (Figure 1). This result may be due to the long residence time of 4 h at 60 °C of the initial larvae sample within the extraction cell during the SFE procedure, which could initiate lipid oxidation, compared to the almost negligible residence time in the case of mechanical pressing, which works under a continuous flow of the larvae within the screw-expeller, although around 135 °C was used in this case. Additionally, another difference is that in the case of SFE, the larvae are previously ground, compared to the whole larvae used for mechanical pressing. Thus, the large exposured surface of the ground particles could favor the oxidation reaction rates or even the contact with potential endogenous pro-oxidant compounds during the time of extraction. Finally, the loss of labile antioxidants or their removal by supercritical CO_2_ could not be ruled out. Therefore, further studies are necessary to understand the impact of SFE defatting of BSFL on the lipid oxidation of the defatted meals, which was not evidenced in the major extracted fat collected from the separator cell of SFE. For example, reducing the heat exposure of the sample during SFE defatting may be possible by increasing the CO_2_ flow/larvae ratio used in our work (0.26 min^−1^), as demonstrated by Cantero-Bahillo et al. (2022) [11] who obtained around 85% fat recovery in just 30 min under the same pressure and temperature conditions (450 bar and 60 °C) but with a CO_2_ flow/larvae ratio in the range of 0.6–1.0 min^−1^.

**Figure 7:**
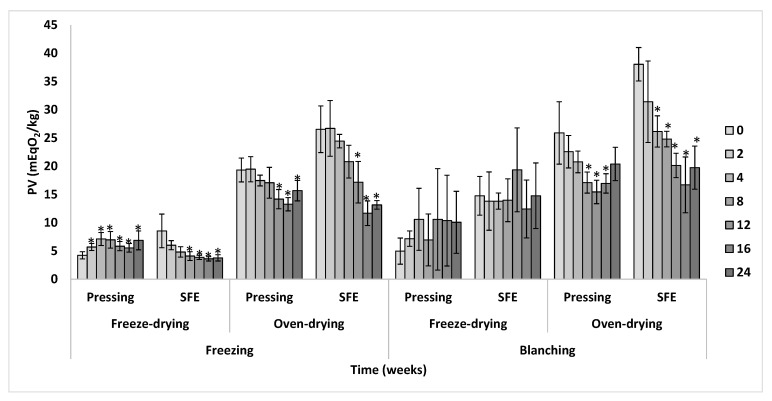
Oxidative stability (PV) during storage of the defatted meals from BSFL processed by different methods of slaughtering, drying, and defatting. Samples with an asterisk within each treatment mean significant difference with respect to its respective initial value at week 0. Each data point corresponds to mean values ± SD, n = 4.

The oxidative deterioration of the defatted meals during 6 months of storage is shown in Figure 7.

One of the most remarkable results was that a general pattern of decreasing PV was observed during the storage following oven-drying, while PV almost did not change after freeze-drying. In general, the typical evolution of hydroperoxides in foods during storage is to increase over time due to the progressive formation reaction of these compounds. However, hydroperoxides are unstable compounds that can simultaneously decompose into secondary oxidation compounds. Therefore, when hydroperoxides tend to decrease with the storage of foods, this suggests that the rate of a decomposition reaction is higher than the rate of a formation reaction; hence the decomposition reactions prevail [39,40]. As this effect was mainly observed in oven-dried samples, it suggests that this thermal processing method may favor such decomposition reaction of hydroperoxides during the storage of these meals. Therefore, it might be expected that these samples would show a higher content of secondary oxidation products, both volatile and non-volatile compounds. Further studies in this sense would be of interest as secondary oxidation reactions can impact the formation of undesirable off-flavors and unhealthy compounds that would compromise the shelf-life of the meals. In this sense, typical methods of secondary oxidation analysis are the p-anisidine value or the measure of thiobarbituric acid reactive substances, both related to volatile compounds of oxidation. However, in the case of edible insects, these parameters have not been almost tested, and reference values to evaluate the magnitude of the results obtained by these methods have not been still recommended for edible insects. Additionally, if we just consider the references of Codex Alimentarius for the oxidative quality of different oils [26,27], secondary oxidation parameters as p-anisidine value, are only recommended for fish oil [26], mainly due to unsaturated fatty acids. In contrast, for the rest of the oils, less unsaturated, as BSFL is, the oxidative quality only measured as PV is recommended in such regulations [27]. Therefore, this parameter has been the only one considered in the current study.

Concerning the effect of the rest of the factors, the same general pattern observed during the storage of the extracted fat (Figure 4) was kept in case of the storage of the defatted meal. Thus, samples obtained from slaughtering by freezing and samples from defatting by mechanical pressing were generally more stable (*p* [slaughtering] = 0.005 and *p* [defatting] < 0.001, respectively, regardless of the time of storage). Additionally, in this case, significant interactions (*p* < 0.05) were observed between slaughtering and drying and between slaughtering and defatting. Therefore, the freeze-dried samples were, in general, the most stable from the PV point of view, especially when derived from blanching, regardless of the defatting mode, as there was no significant evolution of PV during storage. Nevertheless, it should be remarkable that samples obtained through freeze-drying derived from freezing had the lowest values of peroxides throughout the whole storage period.

Previous information on the oxidative stability of BSFL-defatted meal is scarce, and the deep exploration of the impact of the interrelation between the major three processing stages has not been considered. For example, when considering only the slaughtering method, Zhen et al. [19] monitored the evolution of secondary oxidation values by measuring the malondialdehyde content of BSFL meal. They found an increasing trend during the storage period of 28 days, which was higher for freezing than blanching. The initial value at day 0 was also higher for freezing than blanching. The authors explained that the heat treatment of blanching could destroy the enzymes that are involved in lipid oxidation. In the current study, no such clear advantage of blanching over freezing was observed, at least in terms of primary oxidation. In this respect, it is important to take into account that many other factors could be involved in the entire process of slaughtering, drying, and defatting of insects, which may explain the divergences between studies. Thus, for the same method of slaughtering, drying, or defatting, huge different conditions can be used, such as different times and rates of the processes, temperatures, ratios of larvae to water for blanching, and the capacity and power of instruments for freeze-drying or SFE. Moreover, subsequent storage times and storage conditions after processing, among others, can also vary. Therefore, further research is still needed.

## 4. Conclusions

The different processing methods tested for BSFL, such as slaughtering and drying, affect the initial oxidative state of the fat as independent factors, with freezing and freeze-drying as the best methods, respectively. In terms of defatting, mechanical pressing, and SFE produce comparable results, and both methods are much more respectful than conventional hexane defatting. Additionally, interactions take place between the defatting and the slaughtering methods, the defatting, and the drying method, as well as between the three processing steps on the initial oxidative state. Thus, it can be concluded that the combination of freezing, freeze-drying and mechanical pressing could be the most desirable combination of methods to produce the fat with the best initial oxidative quality from the peroxide value point of view. Additionally, regardless of the slaughtering, such a combination of freeze-drying and mechanical pressing also produces the most stable fats after 6 months of storage. In contrast, the combination of blanching and SFE causes the worst stability, regardless of the drying method. It is interesting to remark that such different oxidative stability of BSFL fat during storage seems to be strongly related to the presence of minor antioxidant compounds in the fats, which exhibit an antioxidant activity that is also variable depending on the processing methods of BSFL. On the other hand, the oxidative stability under the conditions of storage does not agree with the stability under accelerated conditions of Rancimat. In this last case, the freeze-dried samples are the least stable, partially related to the amount of free fatty acids. Finally, concerning the oxidative stability of the defatted meals, it follows a generally similar pattern to the extracted fat as affected by the different processing methods, although the oxidation of SFE defatted meals is worse compared to the extracted fat.

Therefore, this study highlights, for the first time, the relevant impact that the main successive processing methods currently used for producing protein-rich products from BSF have on the initial oxidative deterioration of the obtained lipid co-product and protein-rich product. It provides guidance on selecting the most interesting combinations of slaughtering, drying, and defatting procedures for obtaining lipids with optimal initial oxidative quality. Nevertheless, further studies are of interest to investigate the impact of the processing conditions of BSFL on the formation of secondary oxidation compounds to provide a more comprehensive understanding of the entire lipid oxidation process.

## Data Availability

The data are available from the corresponding author.
